# 3-Ethyl­sulfinyl-2-(3-fluoro­phen­yl)-5-iodo-7-methyl-1-benzofuran

**DOI:** 10.1107/S1600536812039463

**Published:** 2012-09-22

**Authors:** Hong Dae Choi, Pil Ja Seo, Uk Lee

**Affiliations:** a Department of Chemistry, Dongeui University, San 24 Kaya-dong Busanjin-gu, Busan 614-714, Republic of Korea; b Department of Chemistry, Pukyong National University, 599-1 Daeyeon 3-dong Nam-gu, Busan 608-737, Republic of Korea

## Abstract

In the title compound, C_17_H_14_FIO_2_S, the 3-fluoro­phenyl ring makes a dihedral angle of 14.56 (5)° with the mean plane [r.m.s. deviation = 0.012 (1) Å] of the benzofuran fragment. In the crystal, mol­ecules are linked *via* pairs of I⋯O contacts [3.038 (2) Å], forming inversion dimers. In the 3-fluoro­phenyl ring, the F atom is disordered over two positions, with site-occupancy factors of 0.747 (3) and 0.253 (3).

## Related literature

For background information and the crystal structures of related compounds, see: Choi *et al.* (2010 ▶, 2011 ▶). For a review of halogen bonding, see: Politzer *et al.* (2007 ▶).
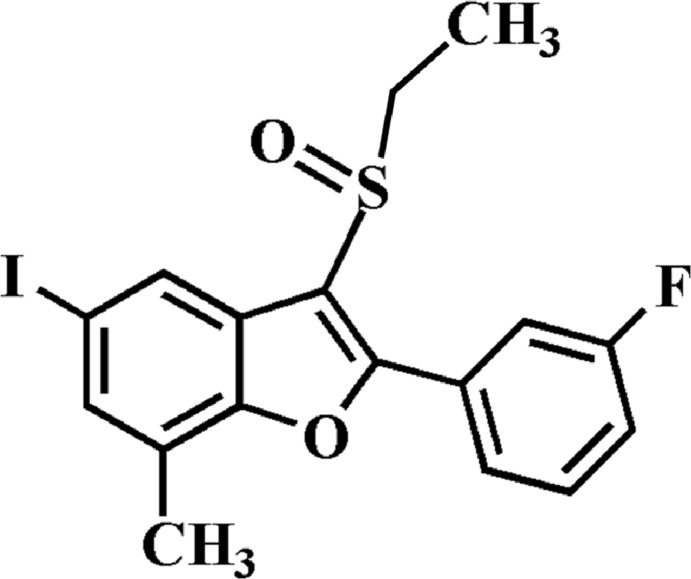


## Experimental

### 

#### Crystal data

C_17_H_14_FIO_2_S
*M* *_r_* = 428.24
Triclinic, 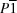

*a* = 7.3338 (2) Å
*b* = 10.3610 (2) Å
*c* = 10.9799 (2) Å
α = 104.644 (1)°
β = 92.926 (1)°
γ = 102.035 (1)°
*V* = 784.71 (3) Å^3^
*Z* = 2
Mo *K*α radiation
μ = 2.19 mm^−1^
*T* = 173 K
0.35 × 0.29 × 0.24 mm

#### Data collection

Bruker SMART APEXII CCD diffractometer
Absorption correction: multi-scan (*SADABS*; Bruker, 2009 ▶) *T*_min_ = 0.556, *T*_max_ = 0.746
14623 measured reflections
3918 independent reflections
3731 reflections with *I* > 2σ(*I*)
*R*_int_ = 0.040

#### Refinement

*R*[*F* ^2^ > 2σ(*F* ^2^)] = 0.022
*wR*(*F* ^2^) = 0.056
*S* = 1.08
3918 reflections
211 parameters
164 restraints
H-atom parameters constrained
Δρ_max_ = 0.38 e Å^−3^
Δρ_min_ = −0.65 e Å^−3^

### 

Data collection: *APEX2* (Bruker, 2009 ▶); cell refinement: *SAINT* (Bruker, 2009 ▶); data reduction: *SAINT*; program(s) used to solve structure: *SHELXS97* (Sheldrick, 2008 ▶); program(s) used to refine structure: *SHELXL97* (Sheldrick, 2008 ▶); molecular graphics: *ORTEP-3* (Farrugia, 1997 ▶) and *DIAMOND* (Brandenburg, 1998 ▶); software used to prepare material for publication: *SHELXL97*.

## Supplementary Material

Crystal structure: contains datablock(s) global, I. DOI: 10.1107/S1600536812039463/aa2071sup1.cif

Structure factors: contains datablock(s) I. DOI: 10.1107/S1600536812039463/aa2071Isup2.hkl

Supplementary material file. DOI: 10.1107/S1600536812039463/aa2071Isup3.cml

Additional supplementary materials:  crystallographic information; 3D view; checkCIF report

## Figures and Tables

**Fig. 1.**
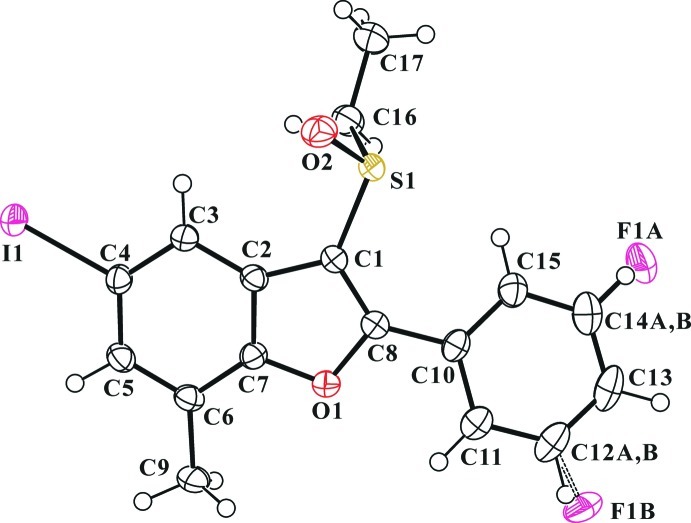
The molecular structure of the title compound with the atom numbering scheme. Displacement ellipsoids are drawn at the 50% probability level. H atoms are presented as small spheres of arbitrary radius. The F atom of the 3-fluorophenyl ring is disordered over two positions with site occupancy factors, from refinement of 0.747 (3) (part A) and 0.253 (3) (part B).

## supplementary crystallographic information

### Comment

As a part of our ongoing study of 3-ethylsulfinyl-5-iodo-7-methyl-1-benzofuran derivatives containing 2-(4-fluorophenyl) (Choi *et al.*, 2010) and 2-(4-chlorophenyl) (Choi *et al.*, 2011) substituents, we report herein the crystal structure of the title compound.

In the title molecule (Fig. 1), the benzofuran unit is essentially planar, with a mean deviation of 0.012 (1) Å from the least-squares plane defined by the nine constituent atoms. In the 3-fluorophenyl ring, the F atom is disordered over two positions with site-occupancy factors of 0.747 (3) (part A) and 0.253 (3) (part B). The dihedral angle between the 3-fluorophenyl ring and the mean plane of the benzofuran ring is 14.56 (5)°. In the crystal structure, molecules are connected by an I···O halogen-bonding between the iodine and the oxygen of the S═O unit [I···O2 = 3.038 (2) Å, C4–I1···O2^i^ = 168.18 (6)°, (i): -*x*, -*y* + 1, -*z*] (Politzer *et al.*, 2007).

### Experimental

3-Chloroperoxybenzoic acid (77%, 224 mg, 1.0 mmol) was added in small portions to a stirred solution of 3-ethylsulfanyl-2-(3-fluorophenyl)-5-iodo-7-methyl-1-benzofuran (330 mg, 0.8 mmol) in dichloromethane (40 ml) at 273 K. After being stirred at room temperature for 3 h, the mixture was washed with saturated sodium bicarbonate solution and the organic layer was separated, dried over magnesium sulfate, filtered and concentrated at reduced pressure. The residue was purified by column chromatography (hexane–ethyl acetate, 2:1 v/v) to afford the title compound as a colorless solid [yield 71%, m.p. 438–439 K; *R*_f_ = 0.53 (hexane–ethyl acetate, 2:1 v/v)]. Single crystals suitable for X-ray diffraction were prepared by slow evaporation of a solution of the title compound in acetone at room temperature.

### Refinement

All H atoms were geometrically positioned and refined using a riding model, with C–H = 0.95 Å for the aryl, 0.99 Å for the methylene, and 0.98 Å for the methyl H atoms. *U*_iso_(H) = 1.2*U*_eq_(C) for the aryl and methylene H atoms, and 1.5*U*_eq_(C) for the methyl H atoms. The positions of methyl and methylene hydrogens were optimized rotationally. The F1 atom of the 3-fluorophenyl ring is disordered over two positions with site occupancy factors, from refinement of 0.747 (3) (part A) and 0.253 (3) (part B). For the proper treatement of H-atoms carbon atoms C12 and C14 were divided in two parts with equalized coordinates and thermal parameters. The distance of equivalent C-F pairs were restrained to 1.330 (5) Å using the SHELXL97 command DFIX, and displacement ellipsoids of F1 set were restrained using the SHELXL97 command ISOR.

### Crystal data

**Table:**

C_17_H_14_FIO_2_S	*Z* = 2
*M**_r_* = 428.24	*F*(000) = 420
Triclinic, *P*1	*D*_x_ = 1.812 Mg m^−^^3^
Hall symbol: -P 1	Mo *K*α radiation, λ = 0.71073 Å
*a* = 7.3338 (2) Å	Cell parameters from 9894 reflections
*b* = 10.3610 (2) Å	θ = 2.4–28.4°
*c* = 10.9799 (2) Å	µ = 2.19 mm^−^^1^
α = 104.644 (1)°	*T* = 173 K
β = 92.926 (1)°	Block, colourless
γ = 102.035 (1)°	0.35 × 0.29 × 0.24 mm
*V* = 784.71 (3) Å^3^	

### Data collection

**Table:**

Bruker SMART APEXII CCD diffractometer	3918 independent reflections
Radiation source: rotating anode	3731 reflections with *I* > 2σ(*I*)
Graphite multilayer monochromator	*R*_int_ = 0.040
Detector resolution: 10.0 pixels mm^-1^	θ_max_ = 28.4°, θ_min_ = 1.9°
φ and ω scans	*h* = −9→9
Absorption correction: multi-scan (*SADABS*; Bruker, 2009)	*k* = −13→13
*T*_min_ = 0.556, *T*_max_ = 0.746	*l* = −14→14
14623 measured reflections	

### Refinement

**Table:**

Refinement on *F*^2^	Primary atom site location: structure-invariant direct methods
Least-squares matrix: full	Secondary atom site location: difference Fourier map
*R*[*F*^2^ > 2σ(*F*^2^)] = 0.022	Hydrogen site location: difference Fourier map
*wR*(*F*^2^) = 0.056	H-atom parameters constrained
*S* = 1.08	*w* = 1/[σ^2^(*F*_o_^2^) + (0.0255*P*)^2^ + 0.2925*P*] where *P* = (*F*_o_^2^ + 2*F*_c_^2^)/3
3918 reflections	(Δ/σ)_max_ = 0.001
211 parameters	Δρ_max_ = 0.38 e Å^−^^3^
164 restraints	Δρ_min_ = −0.65 e Å^−^^3^

### Special details

**Table:**

Geometry. All s.u.'s (except the s.u. in the dihedral angle between two l.s. planes) are estimated using the full covariance matrix. The cell s.u.'s are taken into account individually in the estimation of s.u.'s in distances, angles and torsion angles; correlations between s.u.'s in cell parameters are only used when they are defined by crystal symmetry. An approximate (isotropic) treatment of cell s.u.'s is used for estimating s.u.'s involving l.s. planes.
Refinement. Refinement of F^2^ against ALL reflections. The weighted R-factor wR and goodness of fit S are based on F^2^, conventional R-factors R are based on F, with F set to zero for negative F^2^. The threshold expression of F^2^ > 2sigma(F^2^) is used only for calculating R-factors(gt) etc. and is not relevant to the choice of reflections for refinement. R-factors based on F^2^ are statistically about twice as large as those based on F, and R-factors based on ALL data will be even larger.

### Fractional atomic coordinates and isotropic or equivalent isotropic displacement parameters (Å^2^)

**Table:**

	*x*	*y*	*z*	*U*_iso_*/*U*_eq_	Occ. (<1)
I1	−0.011910 (18)	0.247792 (12)	−0.066263 (10)	0.03114 (5)	
S1	0.27413 (7)	0.74595 (4)	0.42237 (4)	0.02679 (10)	
O1	0.21349 (18)	0.39126 (13)	0.50876 (12)	0.0258 (3)	
O2	0.1125 (2)	0.75365 (15)	0.33857 (14)	0.0336 (3)	
C1	0.2420 (3)	0.57482 (18)	0.43099 (17)	0.0249 (3)	
C2	0.1699 (3)	0.45433 (17)	0.32762 (17)	0.0238 (3)	
C3	0.1203 (3)	0.42768 (18)	0.19759 (17)	0.0254 (3)	
H3	0.1301	0.4996	0.1574	0.030*	
C4	0.0563 (3)	0.29164 (18)	0.13005 (17)	0.0259 (3)	
C5	0.0365 (3)	0.18389 (19)	0.18758 (19)	0.0280 (4)	
H5	−0.0123	0.0925	0.1374	0.034*	
C6	0.0869 (3)	0.20809 (18)	0.31681 (18)	0.0266 (4)	
C7	0.1532 (3)	0.34487 (18)	0.38160 (17)	0.0238 (3)	
C8	0.2659 (2)	0.53213 (18)	0.53774 (17)	0.0244 (3)	
C9	0.0763 (3)	0.0950 (2)	0.3810 (2)	0.0353 (4)	
H9A	0.2031	0.0840	0.4008	0.053*	
H9B	0.0012	0.0092	0.3246	0.053*	
H9C	0.0177	0.1180	0.4595	0.053*	
C10	0.3342 (3)	0.5987 (2)	0.67072 (17)	0.0269 (4)	
C11	0.2991 (3)	0.5240 (2)	0.76036 (19)	0.0317 (4)	
H11	0.2285	0.4318	0.7353	0.038*	
C12A	0.3681 (3)	0.5856 (3)	0.8849 (2)	0.0415 (5)	0.747 (3)
H12A	0.3459	0.5338	0.9449	0.050*	0.747 (3)
C12B	0.3681 (3)	0.5856 (3)	0.8849 (2)	0.0415 (5)	0.253 (3)
F1B	0.3501 (9)	0.5380 (6)	0.9793 (5)	0.0474 (16)	0.253 (3)
C13	0.4679 (3)	0.7192 (3)	0.9258 (2)	0.0439 (5)	
H13	0.5145	0.7603	1.0123	0.053*	
C14A	0.4974 (3)	0.7906 (2)	0.8370 (2)	0.0416 (5)	0.747 (3)
F1A	0.5917 (3)	0.91806 (18)	0.87743 (19)	0.0506 (6)	0.747 (3)
C14B	0.4974 (3)	0.7906 (2)	0.8370 (2)	0.0416 (5)	0.253 (3)
H14B	0.5641	0.8838	0.8639	0.050*	0.253 (3)
C15	0.4356 (3)	0.7346 (2)	0.7108 (2)	0.0341 (4)	
H15	0.4613	0.7874	0.6519	0.041*	
C16	0.4722 (3)	0.7479 (2)	0.3304 (2)	0.0340 (4)	
H16A	0.5780	0.7295	0.3777	0.041*	
H16B	0.4372	0.6750	0.2496	0.041*	
C17	0.5320 (4)	0.8859 (2)	0.3032 (2)	0.0437 (5)	
H17A	0.4294	0.9018	0.2524	0.066*	
H17B	0.6424	0.8872	0.2565	0.066*	
H17C	0.5631	0.9582	0.3833	0.066*	

### Atomic displacement parameters (Å^2^)

**Table:**

	*U*^11^	*U*^22^	*U*^33^	*U*^12^	*U*^13^	*U*^23^
I1	0.03983 (9)	0.02785 (8)	0.02227 (8)	0.00625 (6)	0.00093 (5)	0.00227 (5)
S1	0.0375 (2)	0.01877 (19)	0.0227 (2)	0.00580 (17)	0.00145 (18)	0.00388 (16)
O1	0.0304 (6)	0.0231 (6)	0.0247 (6)	0.0058 (5)	0.0021 (5)	0.0080 (5)
O2	0.0386 (8)	0.0319 (7)	0.0349 (7)	0.0133 (6)	0.0028 (6)	0.0134 (6)
C1	0.0292 (9)	0.0204 (8)	0.0238 (8)	0.0045 (7)	0.0021 (7)	0.0049 (6)
C2	0.0263 (8)	0.0201 (7)	0.0252 (8)	0.0060 (7)	0.0034 (7)	0.0060 (6)
C3	0.0303 (9)	0.0221 (8)	0.0241 (8)	0.0061 (7)	0.0019 (7)	0.0069 (7)
C4	0.0295 (9)	0.0251 (8)	0.0214 (8)	0.0065 (7)	0.0017 (7)	0.0034 (7)
C5	0.0313 (9)	0.0209 (8)	0.0294 (9)	0.0042 (7)	0.0035 (7)	0.0038 (7)
C6	0.0299 (9)	0.0211 (8)	0.0300 (9)	0.0060 (7)	0.0054 (7)	0.0083 (7)
C7	0.0254 (8)	0.0241 (8)	0.0228 (8)	0.0068 (7)	0.0029 (7)	0.0071 (7)
C8	0.0240 (8)	0.0235 (8)	0.0257 (8)	0.0052 (7)	0.0030 (7)	0.0066 (7)
C9	0.0505 (12)	0.0216 (8)	0.0347 (10)	0.0063 (8)	0.0062 (9)	0.0107 (8)
C10	0.0244 (8)	0.0345 (9)	0.0224 (8)	0.0097 (7)	0.0020 (7)	0.0062 (7)
C11	0.0277 (9)	0.0411 (11)	0.0279 (9)	0.0099 (8)	0.0040 (8)	0.0104 (8)
C12A	0.0369 (11)	0.0653 (15)	0.0250 (10)	0.0162 (11)	0.0023 (8)	0.0138 (10)
C12B	0.0369 (11)	0.0653 (15)	0.0250 (10)	0.0162 (11)	0.0023 (8)	0.0138 (10)
F1B	0.060 (3)	0.054 (3)	0.032 (3)	0.006 (3)	0.003 (2)	0.025 (2)
C13	0.0360 (11)	0.0660 (15)	0.0231 (10)	0.0139 (11)	−0.0038 (9)	0.0001 (9)
C14A	0.0360 (11)	0.0438 (12)	0.0362 (12)	0.0075 (10)	−0.0033 (9)	−0.0025 (9)
F1A	0.0606 (13)	0.0339 (9)	0.0430 (11)	−0.0028 (9)	−0.0106 (9)	−0.0012 (8)
C14B	0.0360 (11)	0.0438 (12)	0.0362 (12)	0.0075 (10)	−0.0033 (9)	−0.0025 (9)
C15	0.0345 (10)	0.0348 (10)	0.0290 (10)	0.0050 (8)	−0.0013 (8)	0.0051 (8)
C16	0.0350 (10)	0.0298 (9)	0.0362 (11)	0.0048 (8)	0.0048 (9)	0.0091 (8)
C17	0.0499 (13)	0.0345 (11)	0.0424 (12)	−0.0039 (10)	0.0055 (10)	0.0134 (9)

### Geometric parameters (Å, º)

**Table:**

I1—C4	2.0982 (18)	C9—H9B	0.9800
I1—O2^i^	3.0384 (15)	C9—H9C	0.9800
S1—O2	1.4908 (15)	C10—C15	1.396 (3)
S1—C1	1.7666 (18)	C10—C11	1.401 (3)
S1—C16	1.811 (2)	C11—C12A	1.373 (3)
O1—C7	1.373 (2)	C11—H11	0.9500
O1—C8	1.378 (2)	C12A—C13	1.372 (4)
C1—C8	1.369 (2)	C12A—H12A	0.9500
C1—C2	1.440 (3)	C13—C14A	1.366 (4)
C2—C7	1.393 (2)	C13—H13	0.9500
C2—C3	1.397 (3)	C14A—F1A	1.308 (3)
C3—C4	1.385 (2)	C14A—C15	1.371 (3)
C3—H3	0.9500	C15—H15	0.9500
C4—C5	1.402 (3)	C16—C17	1.515 (3)
C5—C6	1.393 (3)	C16—H16A	0.9900
C5—H5	0.9500	C16—H16B	0.9900
C6—C7	1.385 (3)	C17—H17A	0.9800
C6—C9	1.504 (2)	C17—H17B	0.9800
C8—C10	1.459 (3)	C17—H17C	0.9800
C9—H9A	0.9800		
			
C4—I1—O2^i^	168.17 (6)	H9A—C9—H9C	109.5
O2—S1—C1	107.75 (9)	H9B—C9—H9C	109.5
O2—S1—C16	106.73 (9)	C15—C10—C11	119.02 (18)
C1—S1—C16	97.34 (9)	C15—C10—C8	121.67 (17)
C7—O1—C8	107.04 (13)	C11—C10—C8	119.31 (18)
C8—C1—C2	107.39 (15)	C12A—C11—C10	119.2 (2)
C8—C1—S1	126.73 (14)	C12A—C11—H11	120.4
C2—C1—S1	125.69 (13)	C10—C11—H11	120.4
C7—C2—C3	119.11 (16)	C13—C12A—C11	122.5 (2)
C7—C2—C1	105.07 (15)	C13—C12A—H12A	118.8
C3—C2—C1	135.82 (16)	C11—C12A—H12A	118.8
C4—C3—C2	116.93 (16)	C14A—C13—C12A	117.2 (2)
C4—C3—H3	121.5	C14A—C13—H13	121.4
C2—C3—H3	121.5	C12A—C13—H13	121.4
C3—C4—C5	122.64 (17)	F1A—C14A—C13	116.6 (2)
C3—C4—I1	117.87 (13)	F1A—C14A—C15	120.1 (2)
C5—C4—I1	119.49 (13)	C13—C14A—C15	123.3 (2)
C6—C5—C4	121.33 (17)	C14A—C15—C10	118.8 (2)
C6—C5—H5	119.3	C14A—C15—H15	120.6
C4—C5—H5	119.3	C10—C15—H15	120.6
C7—C6—C5	114.74 (16)	C17—C16—S1	110.13 (16)
C7—C6—C9	122.26 (17)	C17—C16—H16A	109.6
C5—C6—C9	122.97 (17)	S1—C16—H16A	109.6
O1—C7—C6	124.33 (16)	C17—C16—H16B	109.6
O1—C7—C2	110.45 (15)	S1—C16—H16B	109.6
C6—C7—C2	125.21 (17)	H16A—C16—H16B	108.1
C1—C8—O1	110.02 (16)	C16—C17—H17A	109.5
C1—C8—C10	135.75 (17)	C16—C17—H17B	109.5
O1—C8—C10	114.22 (15)	H17A—C17—H17B	109.5
C6—C9—H9A	109.5	C16—C17—H17C	109.5
C6—C9—H9B	109.5	H17A—C17—H17C	109.5
H9A—C9—H9B	109.5	H17B—C17—H17C	109.5
C6—C9—H9C	109.5		
			
O2—S1—C1—C8	−135.41 (17)	C1—C2—C7—O1	−1.2 (2)
C16—S1—C1—C8	114.35 (18)	C3—C2—C7—C6	−1.2 (3)
O2—S1—C1—C2	38.91 (18)	C1—C2—C7—C6	179.34 (18)
C16—S1—C1—C2	−71.33 (18)	C2—C1—C8—O1	0.0 (2)
C8—C1—C2—C7	0.7 (2)	S1—C1—C8—O1	175.20 (13)
S1—C1—C2—C7	−174.51 (14)	C2—C1—C8—C10	179.08 (19)
C8—C1—C2—C3	−178.6 (2)	S1—C1—C8—C10	−5.8 (3)
S1—C1—C2—C3	6.2 (3)	C7—O1—C8—C1	−0.8 (2)
C7—C2—C3—C4	0.1 (3)	C7—O1—C8—C10	179.94 (14)
C1—C2—C3—C4	179.4 (2)	C1—C8—C10—C15	−15.3 (3)
C2—C3—C4—C5	1.5 (3)	O1—C8—C10—C15	163.69 (17)
C2—C3—C4—I1	−178.24 (13)	C1—C8—C10—C11	165.4 (2)
O2^i^—I1—C4—C3	−17.6 (4)	O1—C8—C10—C11	−15.5 (2)
O2^i^—I1—C4—C5	162.6 (2)	C15—C10—C11—C12A	−1.1 (3)
C3—C4—C5—C6	−2.2 (3)	C8—C10—C11—C12A	178.15 (18)
I1—C4—C5—C6	177.54 (14)	C10—C11—C12A—C13	1.2 (3)
C4—C5—C6—C7	1.1 (3)	C11—C12A—C13—C14A	−0.1 (3)
C4—C5—C6—C9	−177.08 (19)	C12A—C13—C14A—F1A	179.6 (2)
C8—O1—C7—C6	−179.30 (17)	C12A—C13—C14A—C15	−1.2 (4)
C8—O1—C7—C2	1.27 (19)	F1A—C14A—C15—C10	−179.6 (2)
C5—C6—C7—O1	−178.80 (16)	C13—C14A—C15—C10	1.3 (3)
C9—C6—C7—O1	−0.6 (3)	C11—C10—C15—C14A	−0.1 (3)
C5—C6—C7—C2	0.6 (3)	C8—C10—C15—C14A	−179.32 (19)
C9—C6—C7—C2	178.76 (18)	O2—S1—C16—C17	67.44 (18)
C3—C2—C7—O1	178.24 (16)	C1—S1—C16—C17	178.52 (17)

Symmetry code: (i) −*x*, −*y*+1, −*z*.
